# WDGTA: a wavelet-denoised graph attention framework for spatial transcriptomics deconvolution

**DOI:** 10.1093/bib/bbag584

**Published:** 2026-10-19

**Authors:** Hao Zhu, Chenggong Ma, Zhenchun Ran, Xumeng Liu, Haobo Tang, Tongquan Wu, Xinglong Wu, Yicheng Xie

**Affiliations:** School of Computer Science and Engineering, Hubei Key Laboratory of Intelligent Robot, Wuhan Institute of Technology, No. 206 Guanggu 1st Road, East Lake High-Tech Development Zone, Wuhan, Hubei 430205, China; School of Computer Science and Engineering, Hubei Key Laboratory of Intelligent Robot, Wuhan Institute of Technology, No. 206 Guanggu 1st Road, East Lake High-Tech Development Zone, Wuhan, Hubei 430205, China; School of Computer Science and Engineering, Hubei Key Laboratory of Intelligent Robot, Wuhan Institute of Technology, No. 206 Guanggu 1st Road, East Lake High-Tech Development Zone, Wuhan, Hubei 430205, China; School of Computer Science and Engineering, Hubei Key Laboratory of Intelligent Robot, Wuhan Institute of Technology, No. 206 Guanggu 1st Road, East Lake High-Tech Development Zone, Wuhan, Hubei 430205, China; School of Computer Science and Engineering, Hubei Key Laboratory of Intelligent Robot, Wuhan Institute of Technology, No. 206 Guanggu 1st Road, East Lake High-Tech Development Zone, Wuhan, Hubei 430205, China; School of Mechanical Engineering, Hefei University of Technology, No. 193 Tunxi Road, Baohe District, Hefei, Anhui 230009, China; School of Computer Science and Engineering, Hubei Key Laboratory of Intelligent Robot, Wuhan Institute of Technology, No. 206 Guanggu 1st Road, East Lake High-Tech Development Zone, Wuhan, Hubei 430205, China; Department of Pulmonology and Genetics and Metabolism, Children's Hospital, Zhejiang University School of Medicine, National Clinical Research Center for Children's and Adolescents' Health and Diseases, No. 3333 Binsheng Road, Binjiang District, Hangzhou, Zhejiang 310052, China; Zhejiang Key Laboratory of Neonatal Diseases, No. 3333 Binsheng Road, Binjiang District, Hangzhou, Zhejiang 310052, China

**Keywords:** spatial transcriptomics, cell-type deconvolution, graph attention network, wavelet denoising, UVR-induced skin aging

## Abstract

Spatial transcriptomics (ST) facilitates the spatial resolution of gene expression profiling; however, many widely used spot-based sequencing platforms capture transcripts from multiple neighboring cells at each location, producing mixed profiles that require accurate cell-type deconvolution. The wavelet-denoised graph attention framework for spatial transcriptomics deconvolution (WDGTA), a graph attention-based framework for ST cell-type deconvolution, was developed and validated on five datasets from two species and four organs. Through the integration of real with pseudo-ST spots, WDGTA introduces a wavelet-based multiscale denoising and reconstruction module, constructs a weighted topological graph using random projection forests, and applies multi-head graph attention to infer cell-type proportions in spatial spots. WDGTA achieved optimal performance in comparison with seven established deconvolution methods. Ablation and discrete wavelet transform (DWT)-specific analyses confirmed the contributions of wavelet denoising, weighted graph construction, and semi-supervised learning. Furthermore, DWT performance remained stable after feature-order perturbation. In mouse brain, cerebellum and ultraviolet radiation (UVR) skin data, WDGTA recovered biologically consistent spatial cell-type patterns and demonstrated robust cross-platform applicability and improved resolution of low-abundance melanocyte signals. Furthermore, it accurately captured melanocyte-associated spatial changes under conditions of UVR exposure and drug intervention. WDGTA provides a unified framework for ST deconvolution across the evaluated tissue contexts. In UVR-exposed skin, it resolved localized melanocyte-associated patterns that were concordant with marker-gene evidence and detected changes associated with UVR exposure and drug treatment. These findings support its use for characterizing spatial cell-type organization and low-abundance cell populations in heterogeneous tissues.

## Introduction

Spatial transcriptomics (ST) has transformed the ability to map gene expression within intact tissue contexts [1]. In contrast to single-cell RNA sequencing (scRNA-seq), ST preserves spatial information, thereby enabling the interpretation of cell states and interactions within their native microenvironment [2]. This spatial context has advanced studies of cell function [3], tissue microenvironments [4], and disease-associated tissue organization, with broad applications in cancer [5], neurological disorders [6], and developmental biology [7]. Recent advances have further extended the application of ST to biologically structured tissues. In the domain of neuroscience, ST has facilitated the investigation of diverse phenomena, including brain development, regional organization, structure–function relationships, and disease-associated spatial alterations [8]. In the domain of skin biology, the integration of single-cell, spatial, and temporal omics has enabled the characterization of tissue organization and cellular remodeling during development, aging, disease, and regeneration [9]. Consequently, ST has been employed in a variety of disease models to map the spatial distribution of disease-associated genes and pathogenic factors [10]. Nevertheless, current ST sequencing technologies have trade-offs in terms of achieving single-cell resolution (10× Genomics Visium platform, Slide-seqV2 [11]) and exhibiting significantly low gene throughput (seqFISH+ [12]). These limitations make it difficult to direct investigation of the mechanisms by which the local microenvironment drives the aging process in specific contexts, such as the skin [13]. Previous studies from our group characterized ultraviolet radiation (UVR)-induced skin inflammation, immunosuppression, and photoaging-related cellular changes, as well as their modulation by Lapagyl and hydroxypinacolone 9-cis retinoate (9-cis HPR) [14, 15]. Conversely, scRNA-seq offers single-cell resolution and extensive transcriptional coverage but lacks spatial coordinates. Therefore, the integration of ST with scRNA-seq is a pivotal strategy for resolving cell-type composition in tissue while retaining spatial context.

In order to infer the spatial distribution of cell types, ST is commonly integrated with scRNA-seq to achieve cell-type deconvolution. Existing deconvolution methods include semi-supervised approaches based on scRNA-seq references [16], reference-free probabilistic models [17], and deep learning frameworks [18]. In related methodological work, our group developed TransAnno-Net, a self-supervised Transformer framework that demonstrated robust cell-type annotation across mouse scRNA-seq datasets and sequencing platforms [19].

In a broader sense, recent task-specific architectures demonstrate the capacity for deep learning to be adapted to address heterogeneous biological prediction problems. For instance, Stacking ENSemble Deep (SENSDeep) combines multiple sequence-based neural networks for the prediction of protein–protein interaction sites [20], and DSRNAFold, a deep-learning-based method for RNA secondary-structure prediction, integrates sequence and structural-context representations for the prediction of RNA secondary structures [21]. While these methodologies address molecular association or structural prediction as opposed to ST deconvolution, they collectively demonstrate the importance of aligning model architecture with the structure and uncertainty of the target biological problem [22].

Deep learning methods have attracted increasing attention because they can model nonlinear relationships and scale to large datasets [23]. These methods often generate pseudo-spots by aggregating scRNA-seq profiles, thereby providing training samples with known cell-type proportions. However, pseudo- and real ST data may exhibit discrepancies in feature distributions due to factors such as sequencing depth, capture efficiency, and batch effects [24]. This discrepancy has the potential to compromise the precision and reliability of cell-type deconvolution. Previous studies have addressed certain aspects of this issue but still have important limitations. For instance, a method for cell-type inference by domain adaptation of single-cell and spatial transcriptomic data (CellDART), mitigates distributional differences through domain adaptation, but it mainly focuses on expression alignment rather than explicitly modeling topological relationships among spatial spots [18]. deconvoluting spatial transcriptomics data through graph-based convolutional networks (DSTG) neural networks into ST deconvolution and demonstrates their utility for estimating cell composition. However, its performance is contingent upon a predefined graph, which may not fully capture complex mappings between pseudo- and real ST data, multiscale spatial correlations, or microenvironmental context in heterogeneous tissues [25].

In order to address these challenges, this study proposes the wavelet-denoised graph attention framework for spatial transcriptomics deconvolution (WDGTA), a graph attention network-based deconvolution framework for integrating pseudo- and real ST data [26]. WDGTA first aligns the two data modalities in a shared feature space and then introduces a wavelet-based multiscale denoising and reconstruction module to obtain more robust expression representations [27]. This module represents the primary methodological innovation of the framework, as it is designed to reduce the influence of sequencing noise, local stochastic variation, and residual batch effects prior to graph construction. Based on the denoised features, WDGTA constructs a global topological graph from spot similarity and uses multi-head graph attention to adaptively aggregate neighborhood information for cell-type composition prediction. WDGTA was evaluated using five quantitative pseudo-ST benchmarks derived from human colorectal, mouse tracheal, and mouse skin scRNA-seq datasets, together with ablation and feature-order sensitivity analyses. The biological applicability of the method was further examined using 10× Genomics Visium mouse brain and UVR-exposed skin data, as well as Slide-seq V2 mouse cerebellum data. Across these applications, WDGTA recovered anatomically concordant cell-type organization and localized melanocyte-associated changes in UVR-exposed skin, supporting its utility for characterizing tissue architecture and spatial responses of low-abundance cell populations across tissue contexts and ST platforms.

## Materials and methods

WDGTA comprised five main phases: data preparation, anchor matching, wavelet-based multiscale denoising and reconstruction, graph construction and training, and performance evaluation (Fig. 1). In the data preparation stage, the integration of annotated scRNA-seq data and real ST data served as the input. Following the implementation of gene filtering, pseudo-ST data were generated by simulating pseudo-spots from annotated scRNA-seq profiles. The pseudo- and real ST data were then mapped into a shared feature space through anchor matching. In the third stage, a wavelet-based multiscale feature denoising and reconstruction module was applied to the integrated expression feature matrix. The denoised and reconstructed feature matrix was subsequently fed into a random projection forest in order to estimate global similarities among spots and construct a weighted topological association graph. Finally, the graph structure and node features were passed into a multi-head graph attention network to predict the cell-type composition of real spatial spots. Model performance was evaluated across a range of both public and private datasets to assess the efficacy and generalizability of WDGTA.

**Figure 1:**
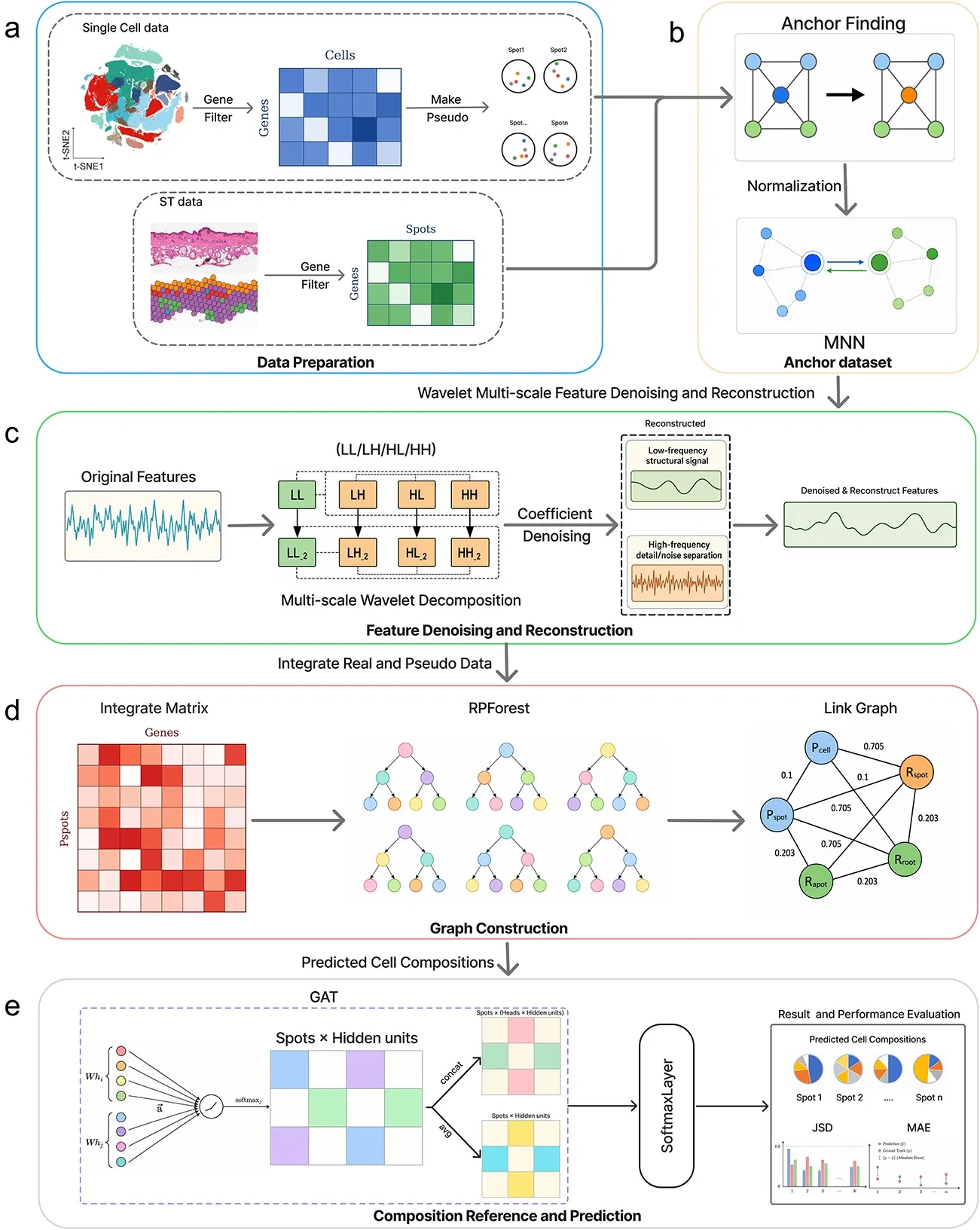
Overview of the WDGTA architecture; (a) data preparation; (b) pseudo- and real ST data are integrated across modalities through anchor matching; (c) integrated expression features are processed using a wavelet-based multiscale denoising and reconstruction module; (d) a weighted topological association graph is constructed; (e) cell-type composition in real ST spots is predicted through adaptive neighborhood aggregation, and deconvolution performance is evaluated.

### Data processing

#### Pseudo-spatial transcriptomics spot construction

To obtain supervised training samples with known cell-type composition labels, pseudo-ST spots were generated from cell-type-annotated scRNA-seq data (Fig. 1a). For each pseudo-spot, 2–10 single cells were randomly sampled, and their gene expression profiles were summed to simulate a mixed spatial spot. The cell-type composition of each pseudo-spot was calculated according to the proportions of the sampled cell types. Thus, the generated pseudo-ST data contained both gene expression profiles and corresponding cell-type proportion labels, and were used as labeled samples for model training, validation, and testing.

#### Cross-modal integration of pseudo- and real spatial transcriptomics data

To reduce data inconsistency between simulated pseudo- and real ST spots, both datasets were normalized prior to cross-modal integration. A mutual nearest neighbor strategy was then employed to identify shared structures between the two modalities and project pseudo- and real ST spots into a unified feature space. The resulting integrated dataset contained both pseudo- and real ST data (Fig. 1b).

### Wavelet-based multiscale feature denoising and reconstruction

Following the cross-modal integration of pseudo- and real ST data, each spatial spot was represented as a high-dimensional expression feature vector. However, the integrated data may still be affected by differences in sequencing depth, uneven capture efficiency, local stochastic variation, and residual batch effects. These factors can introduce unstable noise into the integrated features. Should such noise be directly utilized in graph construction and graph attention propagation, it has the potential to influence the estimation of topological relationships among spots and further compromise cell-type composition prediction. Therefore, a wavelet-based multiscale feature denoising and reconstruction module was incorporated prior to graph construction, with the objective of enhancing the robustness of node features (Fig. 1c).

Specifically, let ${\mathbf{x}}_i\in{\mathbb{R}}^F$ denote the integrated expression feature vector of the *i*-th spatial spot, where *F* represents the input feature dimension. A 1D discrete wavelet transform (DWT) was applied to ${\mathbf{x}}_i$, decomposing it into low-frequency approximation coefficients and high-frequency detail coefficients across multiple scales as follows:


(2-1) \begin{equation*} {\mathbf{x}}_i\overset{\mathrm{DWT}}{\to}\left\{{\mathbf{a}}_i^{(J)},{\mathbf{d}}_i^{(1)},{\mathbf{d}}_i^{(2)},\dots, {\mathbf{d}}_i^{(J)}\right\} \end{equation*}

where ${\mathbf{a}}_i^{(J)}$ denotes the low-frequency approximation coefficients obtained at the *J*-th decomposition level, and ${\mathbf{d}}_i^{(j)}$ denotes the high-frequency detail coefficients at the *j*-th scale.

Subsequent to the acquisition of the multiscale wavelet coefficients, soft-threshold shrinkage was applied to ${\mathbf{d}}_i^{(j)}$ to attenuate unstable high-frequency noise components. Specifically, this approach involved the shrinkage of ${\mathbf{d}}_i^{(j)}$ with small amplitudes, which may be attributed to random noise, to zero while preserving larger-amplitude coefficients that may contain potential biological signals. Accordingly, the reduced high-frequency coefficients ${\tilde{\mathbf{d}}}_i^{(j)}$ at the *j*-th scale were defined as follows:


(2-2) \begin{equation*} {\tilde{\mathbf{d}}}_i^{(j)}=\operatorname{sign}\left({\mathbf{d}}_i^{(j)}\right)\max \left(\left|{\mathbf{d}}_i^{(j)}\right|-{\lambda}_i,0\right),\kern1em j=1,\dots, J \end{equation*}

where ${\lambda}_i$ denotes the threshold for the ${\mathbf{d}}_i^{(j)}$. The noise standard deviation was robustly estimated from the finest-scale detail coefficients using the median absolute deviation, and the threshold was determined using the universal threshold rule as follows:


(2-3) \begin{equation*} {\hat{\sigma}}_i=\frac{{\mathrm{median}}_k\left|{d}_{i,k}^{(1)}\right|}{0.6745},\kern2.00em {\lambda}_i={\hat{\sigma}}_i\sqrt{2}\ln F, \end{equation*}

where *F* denotes the input feature dimension. The same spot-specific threshold was applied to the detail coefficients at all decomposition scales. After that, an inverse discrete wavelet transform (IDWT) was implemented to map ${\mathbf{a}}_i^{(J)}$ and ${\tilde{\mathbf{d}}}_i^{(j)}$ back to the original feature space as follows:


(2-4) \begin{equation*} {\tilde{\mathbf{x}}}_i=\mathrm{IDWT}\left({\mathbf{a}}_i^{(J)},{\tilde{\mathbf{d}}}_i^{(1)},{\tilde{\mathbf{d}}}_i^{(2)},\dots, {\tilde{\mathbf{d}}}_i^{(J)}\right) \end{equation*}

where ${\tilde{\mathbf{x}}}_i\in{\mathbb{R}}^F$ denotes the denoised and reconstructed feature vector of the spot. The denoised and reconstructed features of all spots were combined to form a new node feature matrix *X*, which was used as the input for subsequent random projection tree-based graph construction. The purpose of this module was to reduce the impact of random noise and local stochastic variation on graph topology construction and cell-type composition while preserving key expression information, thereby improving the stability and biological interpretability of the deconvolution results.

### Random projection tree-based graph construction

To capture potential topological relationships among spots in the integrated feature space, a random projection tree-based graph construction strategy was adopted (Fig. 1d). Multiple random projection trees were constructed by means of recursively partitioning the integrated feature matrix. If several spots repeatedly appeared in the same leaf node across multiple projection trees, these spots were considered to have high similarity in the feature space. The leaf-node co-occurrence frequency between different spots across multiple trees was used to define edge weights, thereby constructing a global link graph.

The constructed graph was then symmetrized, and self-loops were added to preserve each node’s own information during graph propagation. In order to reduce noisy connections, edges with weights below a predefined threshold were removed. The resulting adjacency matrix was utilized as the structural input for the graph attention network.

Let *T* denote the number of random projection trees. The edge weight between nodes *i* and *j* was defined as their normalized leaf-node co-occurrence frequency:


(2-5) \begin{equation*} {w}_{ij}=\frac{1}{T}\sum_{t=1}^T\mathbb{I}\left(i\ \mathrm{and}\ j\ \mathrm{occur}\ \mathrm{in}\ \mathrm{the}\ \mathrm{same}\ \mathrm{terminal}\ \mathrm{node}\ \mathrm{of}\ \mathrm{tree}\ t\right) \end{equation*}

Thus, ${w}_{ij}\in \left[0,1\right]$ quantified the stability of the expression-based association between two nodes across the random projection forest. Edges with ${w}_{ij}<0.3$ were removed, and self-loops were assigned ${w}_{ii}=1$. The retained continuous weights were subsequently incorporated into the graph attention calculation rather than being used only to define graph connectivity.

### Model construction

Following the acquisition of the adjacency matrix, a graph attention network was constructed to predict the cell-type composition of spatial spots under graph-structured constraints. Specifically, each spot was treated as a node in the graph. The node features under scrutiny were the integrated expression features that had been reconstructed after wavelet-based denoising. Edges and their weights were defined by the weighted topological association matrix, which encoded the global similarity between spots. The graph attention network adaptively estimated the contribution of different neighboring spots to the cell-type composition inference of a target spot by learning attention coefficients between the target node and its neighbors.

In contrast to graph convolutional methods that employed fixed neighborhood weights or simple average aggregation, this mechanism dynamically adjusted the strength of information propagation according to node expression features and topological connections. This design served to mitigate the impact of noisy neighbors on the prediction, while concurrently enhancing information derived from nodes exhibiting microenvironmental states analogous to the target spot. Finally, the proposed graph attention network obtained the latent representation of each spot through multi-head attention aggregation and generated the corresponding cell-type composition proportions through a Softmax output layer.

#### Calculation of self-attention coefficients

The importance of the features of node ${x}_j$ to node ${x}_i$ was evaluated by introducing a shared attention mechanism. Specifically, the feature representations of the target node and its neighboring node were each linearly transformed by the shared weight matrix W and then concatenated, and the unnormalized attention score ${e}_{ij}$ between target node ${x}_i$ and neighboring node ${x}_j$ was calculated as follows:


(2-6) \begin{equation*} {\mathrm{e}}_{\mathrm{i}\mathrm{j}}=\mathrm{LeakyReLU}\left({\mathrm{a}}^{\mathrm{T}}\left[\mathrm{W}\ast{\mathrm{h}}_{\mathrm{i}}\Big\Vert \mathrm{W}\ast{\mathrm{h}}_{\mathrm{j}}\right]\right) \end{equation*}

where ${h}_i$ and ${h}_j$ denote the feature representations of nodes ${x}_i$ and ${x}_j$, respectively; *W* is a shared learnable weight matrix used for linear feature transformation; *a* is a learnable attention vector; ${a}^T$ denotes the transpose of *a*; denotes vector concatenation, and LeakyReLU is the activation function.

The feature-derived attention score was subsequently modulated by the random projection forest-derived edge weight. For each retained edge $\left(i,j\right)$, the weighted attention score was calculated as:


(2-7) \begin{equation*} {s}_{ij}={w}_{ij}{e}_{ij} \end{equation*}

In order to ensure the comparability of the attention coefficients across different nodes, Softmax normalization was applied exclusively over the first-order neighbors${N}_i$ of node ${x}_i$, as defined by the graph adjacency matrix or mask matrix. The final attention coefficient ${a}_{ij}$ was then calculated as:


(2-8) \begin{equation*} {\alpha}_{ij}=\frac{\exp \left({s}_{ij}\right)}{\sum_{l\in{\mathcal{N}}_i}\exp \left({s}_{il}\right)}=\frac{\exp \left({w}_{ij}{e}_{ij}\right)}{\sum_{l\in{\mathcal{N}}_i}\exp \left({w}_{il}{e}_{il}\right)}, \ \ j\in{\mathcal{N}}_i \end{equation*}

where ${\mathcal{N}}_i$ denotes the set of nodes connected to node *i* after edge pruning, ${w}_{ij}$ is the continuous forest-derived edge weight, ${e}_{ij}$ is the feature-based attention score, and ${\alpha}_{ij}$ is the normalized coefficient used for message aggregation. Consequently, graph topology determined which nodes could exchange information, whereas the continuous edge weights directly modulated the strength of attention assigned to each retained neighbor.

#### Feature aggregation and multi-head attention mechanism

Using the calculated attention coefficients, the proposed network performed a weighted aggregation of neighboring node features and applied the exponential linear unit (ELU) activation function *σ* to update the feature representation of node ${x}_i$:


(2-9) \begin{equation*} \mathop{h_i^{\prime }}\limits^{\rightarrow} =\sigma \left(\mathop\sum\limits_{j\in{\mathcal{N}}_{i}}{\alpha}_{ij}W \mathop{h_j}\limits^{\rightarrow} \right) \end{equation*}

where $\mathop{h_i^{\prime }}\limits^{\rightarrow}$ denotes the updated feature representation of node ${x}_i$, $\sigma \left(\cdotp \right)$ denotes the ELU activation function, ${\alpha}_{ij}$ is the normalized attention coefficient, *W* is the shared learnable weight matrix, and $\mathop{h_j}\limits^{\rightarrow}$ denotes the feature representation of neighboring node ${x}_j$.

In order to stabilize the learning process and capture different space features, a multi-head attention mechanism was adopted. In this model, the number of attention heads was set to 8. In the hidden layer of the network, the outputs of *K* independent attention heads were concatenated as:


(2-10) \begin{equation*} {\left.\mathop{h_i^{\prime }}\limits^{\rightarrow}=\right\Vert}_{k=1}^K{\sigma} \left(\mathop\sum_{j\in{\mathcal{N}}_{\mathcal{i}}}{\alpha}_{ij}^k{\boldsymbol{W}}^{\boldsymbol{k}}\mathop{h_j}\limits^{\rightarrow}\right) \end{equation*}

where *K* denotes the number of attention heads, *k* indexes an individual attention head, $\Big\Vert{}_{k=1}^K$ denotes the concatenation of the outputs from *K* attention heads, and ${\alpha}_{ij}^k$ and ${W}^k$ denote the attention coefficient and learnable weight matrix of the *k*-th attention head, respectively.

It is important to note that a dimensional mismatch may occur in the final output layer of the composition prediction as a result of the conventional concatenation operation. Therefore, averaging was employed as an alternative, and the ELU activation function was removed as follows:


(2-11) \begin{equation*} \mathop{h_i^{out}}\limits^{\rightarrow} =\frac{1}{K}\sum_{k=1}^K\left(\sum_{j\in{\mathcal{N}}_{\mathcal{i}}}{\alpha}_{ij}^k{W}^k\overrightarrow{h_j}\right) \end{equation*}

where $\mathop{h_i^{out}}\limits^{\rightarrow}$ denotes the output representation of node ${x}_i$.

#### Loss function

The training objective was to estimate the cell-type composition of labeled pseudo-ST spots. A masked softmax cross-entropy loss *L* was utilized to ensure that the loss was calculated exclusively over labeled nodes:


(2-12) \begin{equation*} \mathcal{L}=-\sum_{i\in{\mathcal{Y}}_{\mathcal{T}}}\sum_{c=1}^C{Y}_{ic}\log \left({P}_{ic}\right) \end{equation*}

where ${\mathcal{Y}}_T$ denotes the set of labeled pseudo-ST nodes in the training set, *C* is the total number of cell types, ${Y}_{ic}$ denotes the ground-truth proportion of cell type *c* in node *i*, and ${P}_{ic}$ denotes the corresponding proportion predicted by the model. For each node, the ground-truth and predicted proportions range from 0 to 1, and their sum across all cell types is equal to 1. Real ST nodes were excluded from the supervised loss calculation but retained during graph-based information propagation.

#### Spatial transcriptomics cell-type deconvolution

Following model optimization, the selected graph attention network was applied to the weighted topological association graph constructed in the preceding step. The graph contained ${N}_{\mathrm{p}}$ pseudo-ST spot nodes and ${N}_{\mathrm{r}}$ real ST spot nodes. Each node was represented by its denoised and reconstructed expression feature vector, while weighted edges encoded the feature similarity between neighboring nodes. During training, both pseudo-ST spots and real ST spots participated in graph-based information propagation, whereas only pseudo-ST spots with known cell-type proportions contributed to the supervised loss.

The trained model generated a *C*-dimensional cell-type proportion vector for each node through the Softmax output layer. The complete prediction matrix was expressed as follows:


(2-13) \begin{equation*} \hat{\boldsymbol{Y}}=\left[\begin{array}{@{}c@{}}\hat{{\mathrm{Y}}_{\mathrm{p}\mathrm{seudo}}}\\{}\hat{{\boldsymbol{Y}}_{\mathrm{r}\mathrm{eal}}}\end{array}\right]\in{\mathbb{R}}^{\left({N}_{\mathrm{p}}+{N}_{\mathrm{r}}\right)\times C} \end{equation*}

where *Y*, ${\hat{\mathbf{Y}}}_{\mathrm{pseudo}}$, and ${\hat{\mathbf{Y}}}_{\mathrm{real}}$ denote the predicted cell-type proportion matrix for all spots, the predicted proportions for pseudo-spot nodes, and the predicted proportions for real ST spots, respectively. *C* denotes the number of cell types. Therefore, *Y*was retained as the final deconvolution result for downstream cell-type composition analysis and spatial visualization (Fig. 1e).

#### Training settings

The model was trained in accordance with a semi-supervised full graph training strategy [28]. Because the graph attention network propagates information by jointly using node features and topological relationships among nodes, the complete graph was used as the input network in each training iteration, rather than randomly sampling mini-batches of nodes. The full graph contained labeled pseudo-ST spots and unlabeled real ST spots. Real ST spots were not included in the supervised loss calculation, but participated in graph attention propagation. This design enabled the model to use label information from pseudo-ST spots and structural information from unlabeled real ST spots during the training process. Each dataset was randomly divided into training (80%), validation (10%), and test (10%) sets (Supplementary Note S1). In order to verify that the evaluation procedure did not result in information leakage, source-cell identities were audited following data partitioning, and no overlap was detected between the development and held-out evaluation pools. Furthermore, the labels of the held-out nodes were randomly permuted while the node features, graph structure, training labels, model settings, and random seed were kept unchanged (Supplementary Table S1). This particular configuration yielded training and validation trajectories that were indistinguishable, learned model parameters, and final predictions (maximum absolute difference = 0), thereby confirming that neither held-out labels nor overlapping source cells contributed to model optimization (Supplementary Material).

The model was implemented using TensorFlow 2.12 [29], and the parameters were optimized using the Adam optimizer with a cosine warmup decay learning-rate schedule. The upper and lower limits for the learning rate was set at 0.01 and$1\times{10}^{-6}$, respectively. The initial cosine period contained 20 steps, with the warmup phase accounting for 25% of this period. The batch size was set to 50 000 [28], which was larger than the number of nodes in the current graph. Consequently, each training epoch corresponded to one full-graph forward and backward propagation. The maximum number of training epochs was set to 500. Early stopping was used to reduce overfitting. The training process was halted when there was no improvement in the validation loss over successive epochs, and the model parameters with the lowest validation loss were restored.

### Performance evaluation

Pseudo-ST datasets were constructed from a range of scRNA-seq datasets to facilitate the performance evaluation of cell-type deconvolution. As the pseudo-ST data contained known cell-type proportion labels, they were employed as a reference to evaluate the accuracy of WDGTA in estimating cell-type composition at the spot level.

The Jensen–Shannon divergence (JSD) was used to quantify the similarity between two probability distributions [30]. The JSD value ranges from 0 to 1, where 0 indicates that the two distributions are identical and 1 indicates that the two distributions are completely different. In addition, the mean absolute error (MAE) was used as an auxiliary evaluation metric to quantify the absolute deviation between the predicted and true cell-type proportions (Fig. 1e).

The JSD was calculated as follows:


(2-14) \begin{equation*} \mathrm{JSD}=\frac{1}{N}\sum_{i=1}^N\left[\frac{1}{2}\sum_{c=1}^C{y}_{ic}{\log}_2\left(\frac{y_{ic}}{m_{ic}}\right)+\frac{1}{2}\sum_{c=1}^C{\hat{y}}_{ic}{\log}_2\left(\frac{{\hat{y}}_{ic}}{m_{ic}}\right)\right], \end{equation*}

where *N* and *C* denote the number of test spots and cell types, respectively; ${y}_{ic}$ and ${\hat{y}}_{ic}$ denote the normalized reference and predicted proportions of cell type *c* in spot *i*, respectively; and ${m}_{ic}=\left({y}_{ic}+{\hat{y}}_{ic}\right)/2$. Therefore, the JSD ranges from 0 to 1, with lower values indicating greater agreement between the predicted and reference distributions.

The MAE was used as a complementary metric and was calculated as follows:


(2-15) \begin{equation*} \mathrm{MAE}=\frac{1}{NC}\sum_{i=1}^N\sum_{c=1}^C\left|{\hat{y}}_{ic}-{y}_{ic}\right|, \end{equation*}

where *N*, *C*, ${y}_{ic}$, and ${\hat{y}}_{ic}$ are defined as above. The MAE represents the average absolute deviation across all test spot–cell-type pairs, with lower values indicating more accurate cell-type proportion estimates.

### Experimental settings and statistical analysis

WDGTA was implemented in Python 3.9 using TensorFlow 2.12.0. Wavelet processing was performed using PyWavelets 1.7.0, while data preprocessing and numerical analysis were conducted using NumPy 2.0.2, pandas 2.2.2, SciPy 1.31.1, and scikit-learn 1.5.1. The scRNA-seq preprocessing and pseudo-ST spot construction were performed in R 4.3.2 using Seurat 4.5. Model training and evaluation were executed on an Ubuntu 20.04 server equipped with two NVIDIA RTX 4090 graphics processing units (GPUs) and 128 GB of system memory.

### Dataset

The datasets used in this study encompassed two species, mouse and human, and four organs: trachea, colorectal tissue, brain, and dorsal skin. The healthy mouse tracheal scRNA-seq datasets Montoro_10× and Plasschaert were obtained from the Cell BLAST database (https://cblast.gao-lab.org/). The human colorectal cancer scRNA-seq datasets GSE132465 and GSE144735 were obtained from the Gene Expression Omnibus (GEO) database (https://www.ncbi.nlm.nih.gov/geo/). The mouse brain ST dataset (V1_Mouse_Brain_Sagittal_Anterior) was derived from the 10× Genomics repository (https://www.10xgenomics.com/datasets) together with the reference GSE115746. The mouse cerebellum data was derived from the Single Cell Portal under accession SCP948 (https://singlecell.broadinstitute.org/single_cell/study/SCP948). A private dataset on the UVR SKH-1 mouse with dorsal skin scRNA-seq and ST data was provided by the Children’s Hospital affiliated with Zhejiang University (Animal ethics approval: No. ZJU20240193).

The Python source code and R source code for WDGTA have been uploaded to https://github.com/zhss1204-beep/WDGTA.

#### Ultraviolet radiation-exposed skin experimental design and sampling

The UVR-exposed skin data were derived from a previously established SKH-1 mouse photoaging experiment comprising four groups of eight mice each. For the present spatial analysis, one mouse and one independent 10× Genomics Visium skin section were analyzed for each selected condition. No technical replicates were included. The individual mouse was considered the experimental unit, and spatial spots were not treated as independent biological replicates.

## Results

### Performance evaluation

We compared WDGTA with seven representative spatial deconvolution methods, namely CellDART, STRIDE [31], DSTG, Robust Cell Type Decomposition (RCTD) [32], SPOTlight [33], GTAD [34], and SpatialDDLS [35], across five benchmark datasets. For all datasets, quantitative ground-truth proportions were defined by the known cell-type composition of pseudo-ST spots generated from annotated scRNA-seq cells, following established simulation-based benchmarking strategies [36]. All values represent the mean ± standard deviation obtained from five independent runs (Table 1).

**Table 1:** Performance comparison of WDGTA with various methods in five datasets.

Dataset	Species	Organ	Methods	JSD↓	MAE↓
GSE144735	Human	Colorectal	WDGTA (ours)	**0.020 ± 0.002**	0.065 ± 0.007
			CellDART [18]	0.038 ± 0.004	0.072 ± 0.013
			STRIDE [31]	0.026 ± 0.005	0.079 ± 0.024
			DSTG [25]	0.043 ± 0.009	**0.060** ± **0.008**
			RCTD [32]	0.075 ± 0.001	0.068 ± 0.002
			SPOTlight [33]	0.204 ± 0.015	0.134 ± 0.016
			GTAD [34]	0.028 ± 0.012	0.098 ± 0.014
			SpatialDDLS [35]	0.029 ± 0.004	0.071 ± 0.005
GSE132465			WDGTA(ours)	0.124 ± 0.003	**0.074** ± **0.007**
			CellDART	0.157 ± 0.003	0.093 ± 0.014
			STRIDE	0.152 ± 0.007	0.112 ± 0.017
			DSTG	0.214 ± 0.017	0.097 ± 0.019
			RCTD	0.208 ± 0.005	0.076 ± 0.014
			SPOTlight	0.381 ± 0.011	0.163 ± 0.015
			GTAD	0.137 ± 0.012	0.092 ± 0.009
			SpatialDDLS	**0.121 ± 0.002**	0.129 ± 0.012
Montoro_10×	Mouse	Trachea	WDGTA(ours)	**0.128 ± 0.005**	**0.078 ± 0.004**
			CellDART	0.274 ± 0.014	0.121 ± 0.010
			STRIDE	0.284 ± 0.006	0.124 ± 0.007
			DSTG	0.289 ± 0.019	0.088 ± 0.012
			RCTD	0.164 ± 0.007	0.087 ± 0.012
			SPOTlight	0.372 ± 0.035	0.179 ± 0.027
			GTAD	0.138 ± 0.022	0.085 ± 0.012
			SpatialDDLS	0.197 ± 0.002	0.085 ± 0.012
Plasschaert			WDGTA(ours)	0.091 ± 0.002	**0.082 ± 0.002**
			CellDART	0.113 ± 0.012	0.096 ± 0.008
			STRIDE	0.124 ± 0.017	0.093 ± 0.009
			DSTG	0.137 ± 0.009	0.091 ± 0.004
			RCTD	0.126 ± 0.007	0.093 ± 0.010
			SPOTlight	0.192 ± 0.037	0.103 ± 0.028
			GTAD	0.087 ± 0.004	0.104 ± 0.003
			SpatialDDLS	**0.063 ± 0.004**	0.141 ± 0.001
Private		Skin	WDGTA(ours)	**0.291 ± 0.007**	**0.077 ± 0.002**
			CellDART	0.317 ± 0.021	0.121 ± 0.013
			STRIDE	0.316 ± 0.028	0.129 ± 0.021
			DSTG	0.348 ± 0.011	0.092 ± 0.015
			RCTD	0.304 ± 0.017	0.081 ± 0.019
			SPOTlight	0.367 ± 0.045	0.145 ± 0.023
			GTAD	0.304 ± 0.023	0.091 ± 0.019
			SpatialDDLS	0.341 ± 0.001	0.105 ± 0.013

Better results are shown in bold.

In general, WDGTA demonstrated the most robust aggregate performance in comparison to the other evaluated methods, attaining the leading position in seven of the 10 specified dataset-metric combinations. Notably, WDGTA achieved the optimal result for at least one metric on every dataset. WDGTA obtained the lowest mean JSD and MAE on Montoro_10× (0.128 ± 0.005 and 0.078 ± 0.004) and the private skin (0.291 ± 0.007 and 0.077 ± 0.002). In the GSE144735 dataset, WDGTA attained the lowest JSD, while DSTG achieved the lowest MAE. On GSE132465 and Plasschaert, SpatialDDLS yielded lower JSD values, whereas WDGTA achieved the lowest MAE (Table 1).

JSD was utilized to assess the agreement between the predicted and reference cell-type composition distributions at each location, while MAE was employed to quantify the average cell-type-wise deviation. The resultant split rankings are thus indicative of metric-specific trade-offs, rather than consistent underperformance by WDGTA. While DSTG and SpatialDDLS exhibited benefits for specific metrics, WDGTA maintained a complementary advantage in each corresponding dataset and attained the highest number of top-ranked results overall.

It is noteworthy that WDGTA achieved the top ranking for both metrics on the private skin-derived pseudo-ST benchmark, surpassing the second-ranked RCTD in both JSD and MAE. This result extended its strong aggregate performance to a tissue context distinct from the public colorectal and tracheal datasets. Collectively, these findings highlighted the robust performance of WDGTA across diverse datasets and further supported its ability to accurately reconstruct cell-type compositions.

When considered as a whole, the analyses of pseudo-ST cell mixtures and comparative benchmarks identified WDGTA as the most consistently high-performing method across the evaluated datasets. Despite the fact that individual methods demonstrated metric-specific advantages, WDGTA achieved the highest number of first-place results and was placed first for at least one metric on every dataset. The findings provide quantitative support for the robustness of WDGTA across diverse tissue contexts and its ability to accurately reconstruct cell-type compositions.

### Spatial distribution analysis of mouse brain cells

The mouse cerebral cortex is characterized by a highly organized and well-defined laminar cellular architecture (Fig. 2b), which renders it an appropriate subject for the evaluation of spatial deconvolution algorithms in terms of accuracy and sensitivity.

**Figure 2:**
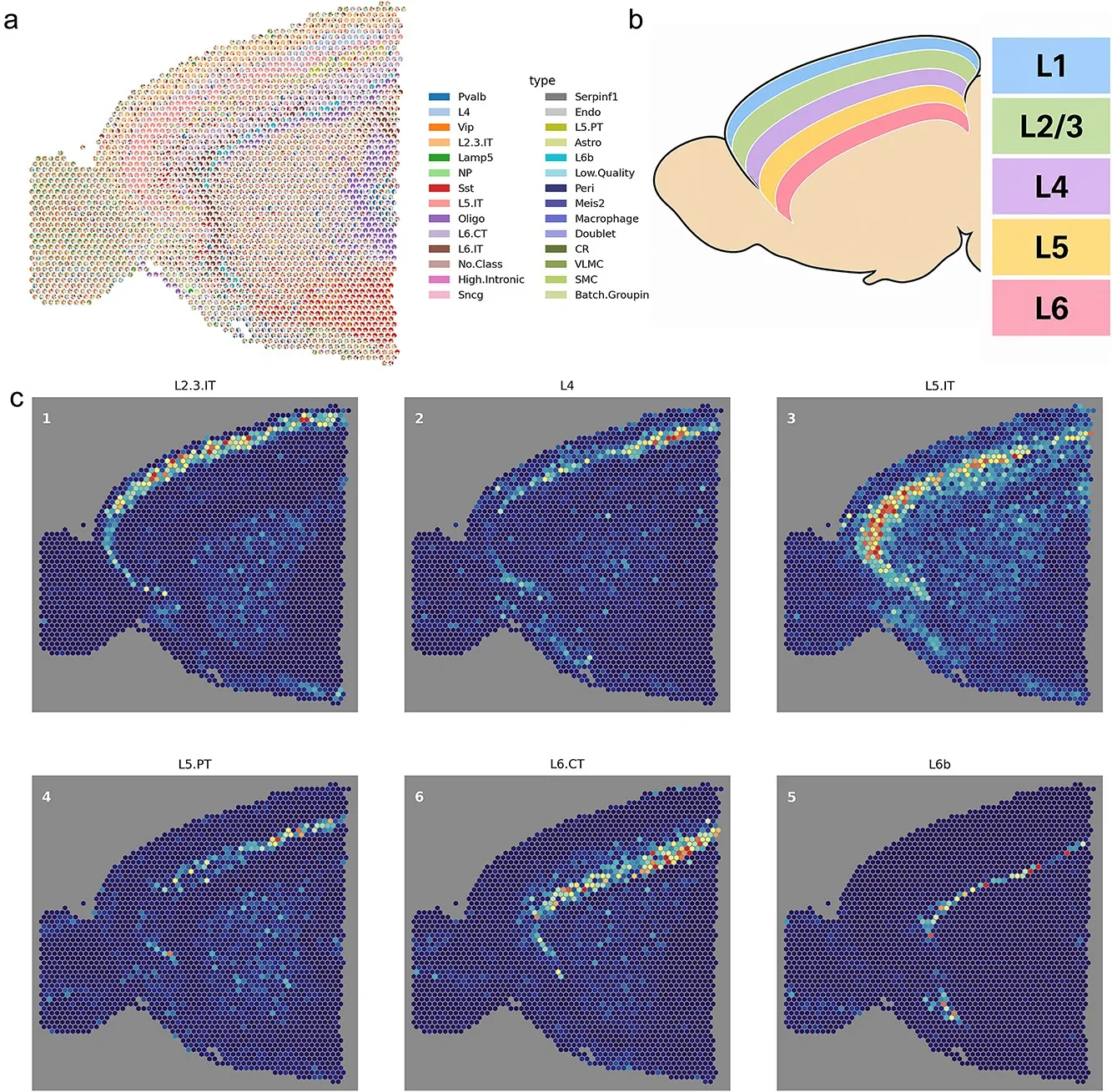
Cell-type composition of mouse cerebral cortex using WDGTA; (a) pie charts showing the predicted cell-type composition of each spot in the cortex; (b) previously reported layer-specific neuronal organization in the cerebral cortex [37]; (c) spatial distribution of six layer-specific excitatory neuronal populations in the mouse cortex.

Firstly, WDGTA performed global spatial deconvolution of the entire mouse brain section with the predicted proportions of all cell subtypes visualized at each spatial spot (Fig. 2a). Secondly, in order to evaluate the model’s ability to resolve fine-scale microenvironments, layer-specific excitatory neurons with strong spatial heterogeneity in the cortical region were examined. WDGTA predicted and spatially mapped the abundance of each neuronal subtype (Fig. 2c). Six cortical excitatory neuronal subtypes, including L2/3, L4, L5 IT, L5 PT, L6b, and L6 CT, exhibited clear and largely non-overlapping band-like spatial distributions across the tissue section. The spatial abundance gradients under scrutiny were found to be consistent with the established laminar organization and transcriptomic taxonomy of excitatory neuronal populations in the adult mouse neocortex (Fig. 2b) [37]. For instance, the L2/3 subtype was predominantly present in the outer superficial cortical layers, while the L6b and L6 CT subpopulations were largely confined to the deepest cortical layers adjacent to the white matter. Overall, WDGTA demonstrated the capability to characterize extensive cellular heterogeneity at the whole-tissue scale and capture fine laminar structures and subtype-specific spatial localization with high sensitivity.

### Ablation study

In order to assess the contribution of the principal components of WDGTA, a comparison was made between the complete model and three ablated variants. In these variants, wavelet-based multiscale denoising, weighted graph construction, or semi-supervised graph learning had been disabled individually. The experiments were conducted on the private UVR-exposed skin, Montoro_10×, and GSE132465 datasets under otherwise identical settings (Table 2). The WDGTA model, in its entirety, consistently demonstrated the lowest JSD and MAE across all three datasets. Conversely, the removal of any individual component resulted in a reduction in performance, thereby supporting the complementary contributions of the three modules.

**Table 2:** Ablation study of WDGTA architecture.

Model	Dataset	Wavelet	Graph	Semi-supervised	JSD ↓	MAE ↓
WDGTA	Private	✓	✓	✓	**0.291**	**0.077**
		✗	✓	✓	0.312	0.089
		✓	✗	✓	0.324	0.093
		✓	✓	✗	0.401	0.127
	Montoro_10×	✓	✓	✓	**0.128**	**0.078**
		✗	✓	✓	0.138	0.084
		✓	✗	✓	0.201	0.124
		✓	✓	✗	0.214	0.135
	GSE132465	✓	✓	✓	**0.124**	**0.074**
		✗	✓	✓	0.137	0.092
		✓	✗	✓	0.144	0.103
		✓	✓	✗	0.132	0.089

Better results are shown in bold.

The removal of the wavelet-based denoising module resulted in a modest but consistent deterioration across datasets, indicating that multiscale denoising contributes to stable cell-type proportion estimation. The elimination of the weighted graph construction resulted in a more pronounced decline in performance on Montoro_10× and GSE132465, which is consistent with the importance of preserving informative relationships among neighboring spots. It is noteworthy that the disablement of semi-supervised graph learning resulted in the most significant performance decline on the private UVR skin and Montoro_10× datasets. In contrast, its impact was less evident on GSE132465, where weighted graph construction emerged as the more salient factor. This dataset-dependent pattern indicates that the relative contribution of each module varies across biological contexts, while their combination provides the most consistent overall performance.

In order to further evaluate the contribution and feature-order sensitivity of the wavelet-based denoising and reconstruction module, a comparison was made across runs of three configurations: original-order wavelet reconstruction, shuffled-order wavelet reconstruction, and removal of the wavelet module. The original-order configuration demonstrated the optimal overall performance, with a JSD of 0.2925, an MAE of 0.0779, and a Pearson correlation of 0.3390. The random re-ordering of the features prior to wavelet decomposition resulted in only a marginal alteration, yielding a JSD of 0.2970, an MAE of 0.0788, and a Pearson correlation of 0.3298. Conversely, the elimination of the wavelet module resulted in a substantial deterioration in performance, as evidenced by an increase in the JSD and MAE to 0.3991 and 0.1090, respectively, while concomitantly leading to a reduction in the Pearson correlation to 0.2134 (Table 3). The findings suggest that the sequence of features had a negligible impact on the reconstructed representation, yet it failed to account for the considerable enhancement in performance that was concomitant with wavelet processing. The findings of the present study lend support to the practical importance of wavelet-based denoising and reconstruction for the purpose of stabilizing node features and improving downstream deconvolution performance. Furthermore, the results reveal a modest residual dependence on feature order.

**Table 3:** Contribution and feature-order sensitivity of the wavelet-based denoising and reconstruction module in WDGTA on the private UVR mouse skin.

Model	Wavelet	Feature order	JSD ↓	MAE ↓	Pearson *r* ↑
WDGTA	✓	Original	**0.2925**	**0.0779**	**0.3390**
	✓	Random	0.2970	0.0788	0.3298
	✗	N.A.	0.3991	0.1090	0.2134

Bold values indicate the best result for each metric (lower JSD and MAE; higher Pearson *r*).

### Application of WDGTA on slide-seq V2 mouse cerebellum data

In order to evaluate the applicability of WDGTA to ST data generated using a different sequencing platform, the model was applied to a publicly available Slide-seq V2 mouse cerebellum dataset [38]. The mouse cerebellum displays a well-defined laminar architecture and cell populations that are confined to specific regions, thus providing an anatomically interpretable framework for evaluating cell-type resolution performance. An annotated mouse cerebellum snRNA-seq dataset was utilized as the reference, and WDGTA was subsequently employed to map the reference-defined cell types to individual spatial beads in the Slide-seq V2 section (Fig. 3a).

**Figure 3:**
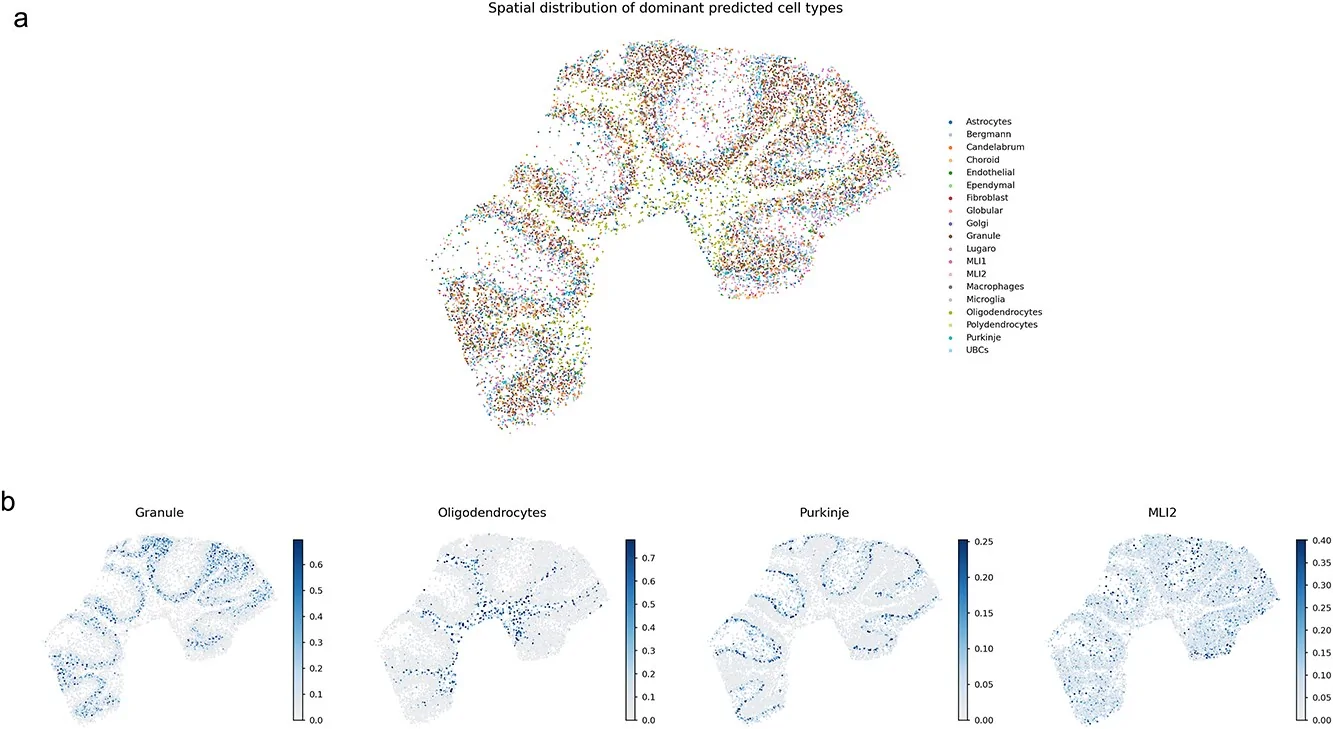
Cross-platform application of WDGTA to slide-seq V2 mouse cerebellum data; (a) spatial distribution of the dominant cell type predicted by WDGTA for each slide-seq V2 bead; each point is representative of a single spatial bead, and is colored according to the cell type for which the highest predicted proportion is recorded among the 19 reference-defined cell populations; (b) spatial distributions of the predicted proportions of granule cells, oligodendrocytes, Purkinje cells, and MLI2; the color intensity of each bead thus serves as an indicator of the predicted proportion of the corresponding cell type.

The mouse cerebellar cortex is comprised of three principal layers: the outer molecular layer, the intermediate Purkinje cell layer, and the inner granular layer. The white matter is located beneath the cortex [39]. The spatial distributions predicted by WDGTA were consistent with this established organization (Fig. 3b and Table 4). Granule cells formed densely enriched, curved domains corresponding to the granular layer across the cerebellar folia. Furthermore, the results of the study indicated that Purkinje-cell signals were concentrated along narrow intermediate trajectories at the boundary between the granular and molecular layers. The distribution of molecular layer interneuron subtype 2 (MLI2) cells was found to be preferential in the outer cortical regions corresponding to the molecular layer, while oligodendrocytes were found to be enriched in deeper regions associated with cerebellar white matter.

**Table 4:** Correspondence between the established cellular organization of the mouse cerebellum and the spatial patterns predicted by WDGTA.

Anatomical region	Expected cell populations	Spatial pattern recovered by WDGTA
Molecular layer	MLI1 and MLI2	MLI2 signals were preferentially distributed along the outer cortical regions of the cerebellar folia
Purkinje cell layer	Purkinje cells and Bergmann glia	Purkinje-cell signals formed narrow intermediate trajectories between the molecular and granular layers
Granular layer	Granule cells	Granule-cell signals formed dense, curved domains following the cerebellar folia
White matter	Oligodendrocytes and astrocytes	Oligodendrocyte signals were enriched in the deeper regions beneath the cerebellar cortex

To provide a structured comparison with the established anatomy of the mouse cerebellum, the expected locations of the major cerebellar cell populations and the corresponding spatial patterns recovered by WDGTA are summarized in Table 3.

Collectively, WDGTA recovered the characteristic laminar organization of the mouse cerebellum, including granule-cell enrichment in the granular layer, Purkinje-cell localization in the intermediate Purkinje cell layer, MLI2 enrichment in the molecular layer, and oligodendrocyte localization within the white matter. The concordance between these predicted distributions and the previously reported cellular architecture of the mouse cerebellum [18] supported the applicability of WDGTA to high-resolution Slide-seq V2 data and its capacity to preserve biologically meaningful spatial organization across distinct tissue contexts and ST platforms.

### WDGTA efficiently reconstructs of spatial cell-type composition in ultraviolet radiation-exposed skin

Through manual annotation of raw spatial sections, major cell types were identified, including adipocytes, basal cells, dendritic epidermal T cells, fibroblasts, keratinocytes, and muscle cells, which served as a reference for the underlying tissue structure (Fig. 4a). Different cell types exhibited clear spatial stratification within skin sections. For instance, keratinocytes and basal cells were predominantly distributed in epidermis-associated regions, whereas fibroblasts were primarily located in the dermal region.

**Figure 4:**
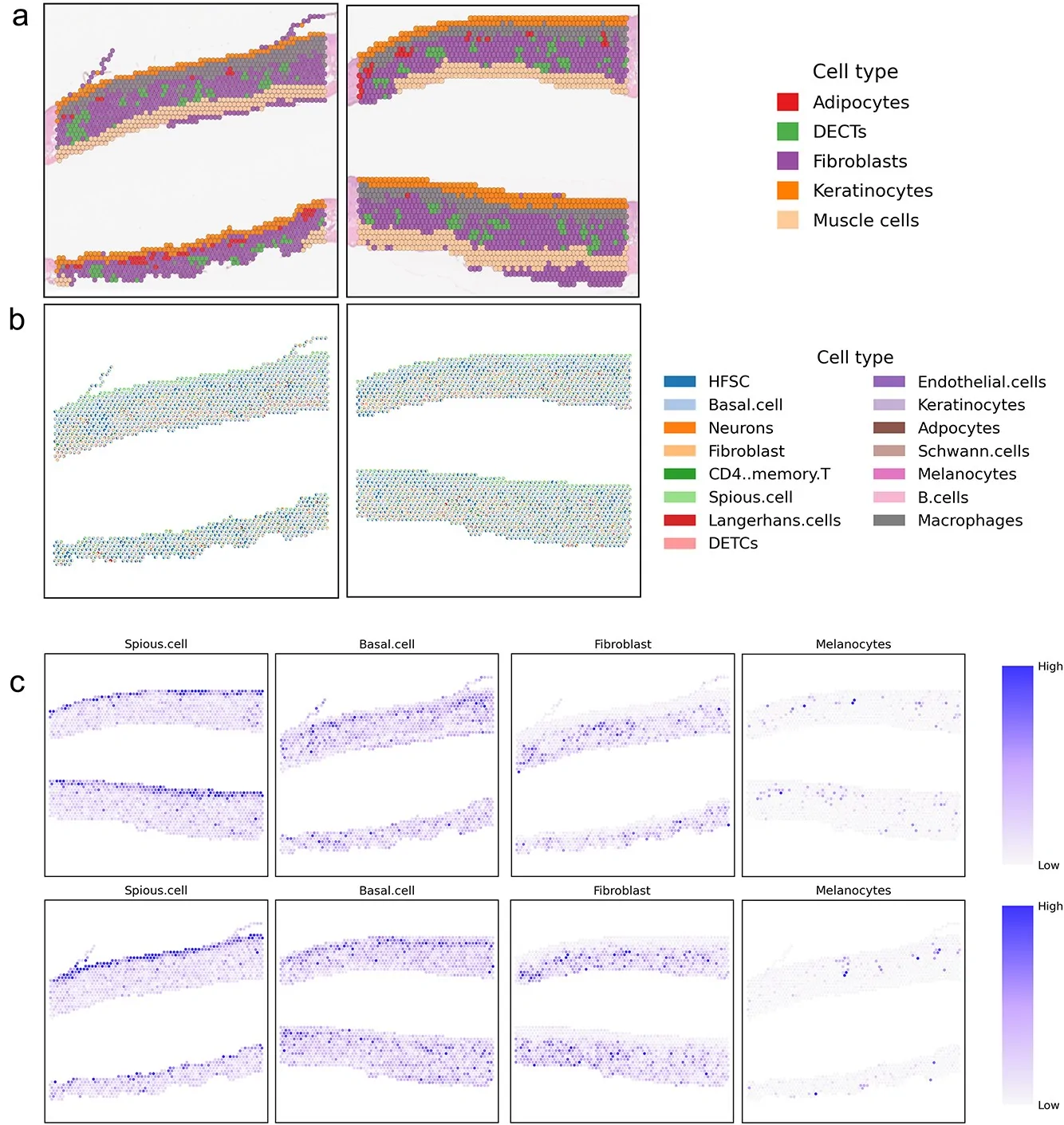
WDGTA reconstructed the spatial distribution of major cell types in UVR-exposed skin; (a) manually annotated cell-type composition maps of two tissue sections; in the left, the upper and lower panel shows the model group and the control group, respectively; in the right, the upper and lower panel shows the retinol-treated group and the low-dose 9-cis HPR-treated group, respectively; (b) spatial deconvolution composition maps generated by WDGTA, displaying the reconstructed spatial organization of major cell types; (c) heatmaps of key cell-type proportions used for validation.

WDGTA then proceeded to reconstruct the relative composition of multiple cell types at the spot level, including hair follicle stem cells (HFSCs), basal cells, and fibroblasts, while preserving the epidermal-dermal layered structure of skin tissue (Fig. 4b). To further illustrate the cell-type decomposition of UVR-exposed skin predicted by WDGTA, proportion heatmaps of representative cell types were visualized, including fibroblasts, spinous cells, basal cells, and melanocytes (Fig. 4c). The distribution of fibroblast signals was found to be predominantly concentrated in the dermal region, a finding that was consistent with the distribution of fibroblasts in the manual annotations. Spinous and basal cells exhibited stronger signals in regions associated with the epidermis. This distribution was consistent with the findings of previous single-cell studies, which demonstrated that mouse epidermal heterogeneity is organized along differentiation-associated and spatial transcriptional axes [40]. Melanocytes demonstrated a relatively sparse distribution, mainly manifesting as localized low-abundance signals. These single-cell-type proportion heatmaps corroborated the hypothesis that WDGTA could recover overall cell-type composition and spatially localize major skin cell types. Collectively, these results suggested that WDGTA could accurately recover the spatial distribution of major cell types in real skin tissue with reliable biological consistency.

### WDGTA accurately characterizes the spatial distribution of melanocytes in ultraviolet radiation-exposed skin

WDGTA and SPOTlight were provided with the same annotated scRNA-seq dataset, in which melanocytes were represented. Consequently, melanocytes were incorporated into the candidate cell-type space available to each method. However, the presence of these cells in the reference did not guarantee accurate recovery of the spatial data, particularly because signals from low-abundance populations may be sparse or masked by more abundant cell types. Consequently, the present comparison concentrated on the spatial accuracy of melanocyte localization as opposed to the ability of each method to merely return melanocytes.

In the context of sequencing-based ST data, spatial concordance between inferred cell-type abundance and the expression of corresponding marker genes has emerged as a prevalent biological plausibility assessment approach. A comprehensive benchmarking study explicitly evaluated deconvolution methods by calculating the correlation between predicted cell-type proportions and marker-gene expression in real spatial datasets [36]. In a similar manner, STdeconvolve evaluated the inferred cell types by comparing their spatial distributions with the corresponding marker genes and *in situ* hybridization images from the Allen Brain Atlas [41]. Marker-gene and *in situ* hybridization patterns have also been used to support spatial cell-type annotation and localization in high-resolution ST studies [42].

In order to evaluate the ability of WDGTA to resolve low-abundance cell types spatially, melanocytes were used as a representative example. The WDGTA predictions were compared with SPOTlight and with a melanocyte module score constructed from dopachrome tautomerase (Dct), premelanosome protein (Pmel), and tyrosinase-related protein 1 (Tyrp1). The three marker genes exhibited sparse and locally enriched spatial expression patterns, consistent with the distributional characteristics of melanocytes as a low-abundance cell type (Fig. 5b). Across the control, UVR, and UVR + drug treatment groups, the overall spatial distributions showed that melanocyte hotspots predicted by WDGTA were more consistent with high module-score regions. In contrast, SPOTlight produced more broadly distributed prediction signals and showed background diffusion in some regions, which was less consistent with the low-abundance nature of melanocytes (Fig. 5a). Local magnified comparisons further showed that, under UVR exposure and UVR + drug treatment conditions, the hotspot locations predicted by WDGTA corresponded well to the marker module score, whereas SPOTlight detected more dispersed signals (Fig. 5c).

**Figure 5:**
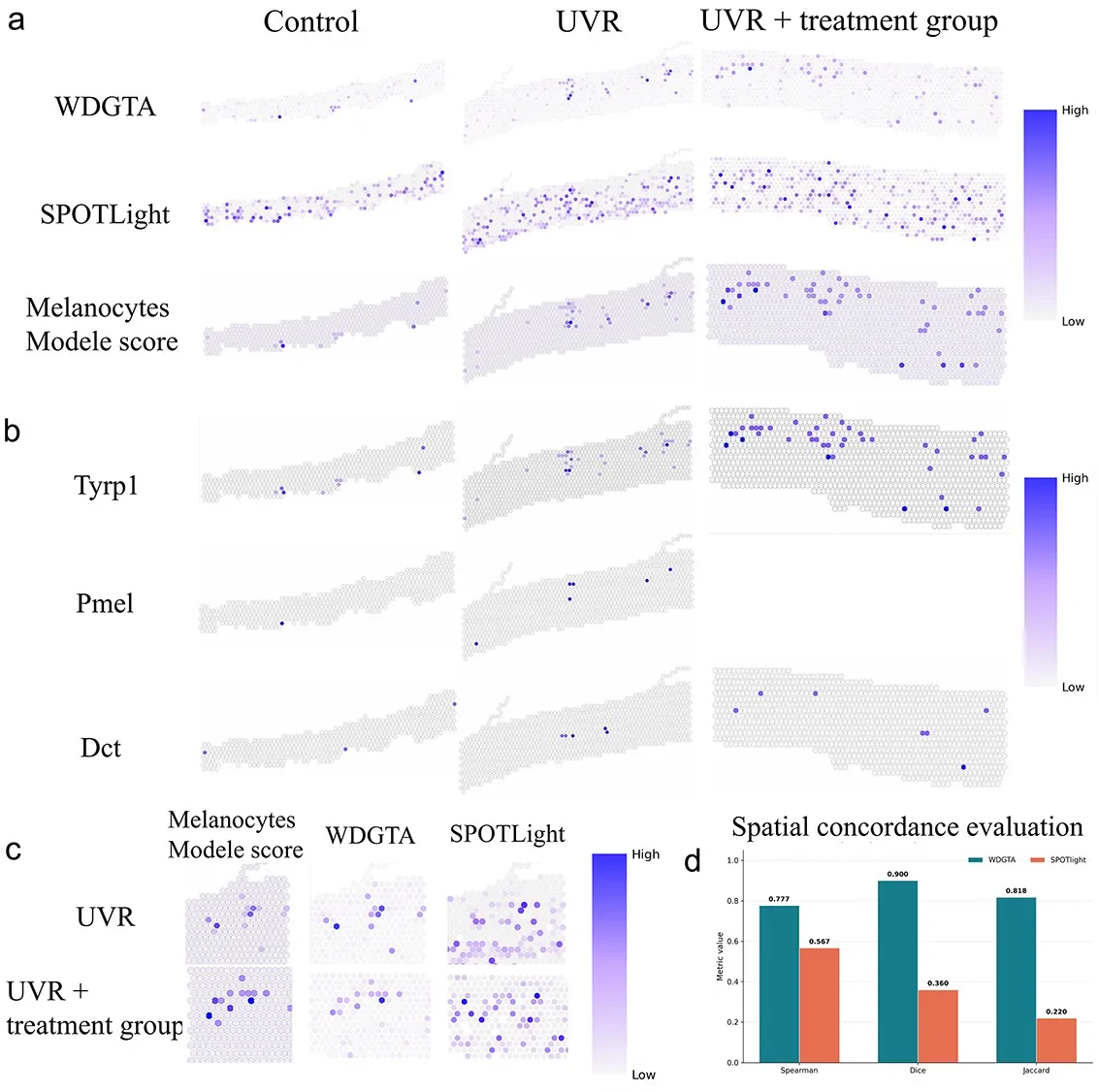
Comparison of WDGTA and SPOTlight in the spatial localization of melanocyte markers in UVR-exposed skin; (a) comparison of the spatial distributions predicted by WDGTA and SPOTlight with melanocyte-associated spatial patterns under the control, UVR, and UVR + drug treatment conditions; (b) spatial visualization of three melanocyte marker genes; no Pmel expression was detected in the UVR treatment group; (c) representative locally magnified regions in the UVR and UVR + drug treatment groups; (d) spatial concordance evaluation with the melanocyte marker module score, including spearman correlation, dice coefficient, and Jaccard index, compared with SPOTlight.

It has been demonstrated that ultraviolet irradiation exerts a stimulatory effect on melanogenesis, which is attributable to both melanocyte-intrinsic responses and keratinocyte-derived melanogenic factors [43]. This established response lends support to the biological plausibility of the augmented melanocyte-associated signals and spatial hotspots that were detected by WDGTA in the UVR group relative to the control group, whereas SPOTlight did not clearly capture melanocyte changes associated with the pathological process (Fig. 5a). The alterations in the melanocyte spatial distribution detected by WDGTA were consistent with pathological features of UVR-induced epidermal hyperplasia and abnormal keratinization [44]. These findings suggest that WDGTA may be capable of identifying UVR-induced tissue injury and microenvironmental changes with greater precision than SPOTlight [45].

In the UVR + drug treatment group, a reduction in melanocyte prediction signals and marker gene hotspots was observed in comparison with the UVR group. This direction of change was consistent with the results of previous single-cell and ST characterization of the SKH-1 photoaging model by our group. In this model, 9-cis HPR was found to downregulate melanogenesis-related genes, including Tyr, Dct, and Tyrp1, as well as melanosome-associated genes such as Pmel [14]. This finding suggests an altered melanocyte-associated spatial pattern following treatment. Conversely, SPOTlight yielded more dispersed prediction signals and lacked the capacity to distinctly replicate these group-level alterations. WDGTA retained the capacity to detect melanocyte-related spatial changes under the treatment condition, indicating a more pronounced ability to discern low-abundance cell types in varied experimental settings (Fig. 5a).

In order to provide further support for this observation, a comparison was made between WDGTA and SPOTlight using metrics such as Spearman correlation, Dice coefficient, and Jaccard index (Fig. 5d). WDGTA achieved values of 0.777, 0.900, and 0.818, respectively, which were higher than those of SPOTlight (0.567, 0.360, and 0.220). The results indicated that WDGTA could reduce spatial displacement and background diffusion in melanocyte prediction, thereby improving the spatial concordance between low-abundance cell-type predictions and marker gene evidence.

## Discussion and conclusion

This study proposed WDGTA, a ST deconvolution framework designed to address distributional discrepancies between pseudo- and real ST data, expression noise, and the difficulty of modeling complex relationships among spots.

In comparison with representative deep learning-based deconvolution methods such as DSTG [25] and CellDART [18], WDGTA integrates wavelet-based multiscale feature denoising and reconstruction, random projection forest-based weighted graph construction, and graph attention-based adaptive neighborhood aggregation within a unified framework. Following cross-modal integration, the wavelet module stabilizes the feature representations used for graph construction, the random projection forest estimates global similarities among spots to construct a weighted topological association graph, and the graph attention network adaptively determines the contributions of neighboring spots during cell-type composition inference.

The purpose of this coordinated design is threefold: first, to reduce residual expression noise, second, to improve the representation of topological relationships, and third, to avoid uniform information propagation across neighbors. As demonstrated by the results of the benchmark evaluations, WDGTA achieved the best or near-best performance across the evaluated datasets. Furthermore, the performance deterioration that was observed after the removal of wavelet denoising, graph construction, or semi-supervised graph learning supported the complementary contributions of these components. The findings, taken collectively, lend support to the applicability of WDGTA to cell-type composition estimation and the characterization of tissue spatial organization and microenvironmental states [46].

Across the five quantitative benchmark datasets, which comprised human colorectal, mouse tracheal, and mouse skin, WDGTA ranked first in seven of the 10 dataset–metric combinations and achieved the lowest mean value for at least one metric in every dataset. The findings indicated robust aggregate performance rather than universal superiority, as DSTG and SpatialDDLS retained metric-specific advantages on individual datasets.

In addition to the quantitative benchmarks, WDGTA was applied to 10× Genomics Visium mouse cerebral cortex and UVR-exposed skin data, as well as Slide-seq V2 mouse cerebellum data. In the cerebral cortex, WDGTA recovered the established laminar distributions of excitatory neuronal populations [37]. In the cerebellum, the predicted distributions of granule cells, Purkinje cells, MLI2 cells, and oligodendrocytes were consistent with the known cerebellar architecture [39]. The results of this study, when considered in conjunction with the preserved epidermal–dermal organization observed in the skin sections, lend further support to the applicability of WDGTA across distinct tissues and ST platforms.

The wavelet-based denoising and reconstruction module constituted a significant component of WDGTA. The elimination of this module resulted in a consistent enhancement of JSD and MAE across the evaluated datasets, suggesting that wavelet processing contributes to the enhancement of downstream deconvolution performance. As 1D DWT may be susceptible to the influence of feature ordering, an additional sensitivity analysis was conducted. This involved randomly permuting the input features prior to wavelet decomposition. The feature permutation produced only modest numerical changes, whereas the complete removal of the wavelet module resulted in substantially greater performance deterioration. These observations indicated that the observed enhancement was not exclusively attributable to a specific feature arrangement, although a modest residual order dependence persisted.

Recent studies have emphasized the importance of interpretability in models when conducting ST analysis. For instance, xSiGra integrates gene expression, spatial coordinates, and histological images through a hybrid graph transformer and applies graph Grad-CAM to quantify the contributions of individual genes and cells to spatial cell-type identification [47]. Although the primary focus of xSiGra is single-cell spatial recognition rather than spot-level cell-type proportion deconvolution, its feature-attribution strategy provides a complementary perspective to WDGTA. The incorporation of gene-level attribution and multimodal tissue information has the potential to enhance the biological interpretability of WDGTA predictions.

In UVR-exposed skin, WDGTA resolved an increase in melanocyte-associated signals in the UVR group relative to the control group. These signals exhibited spatial concordance with Dct, Pmel, Tyrp1, and the corresponding melanocyte module score. WDGTA also demonstrated higher Spearman correlation, Dice coefficient, and Jaccard index than SPOTlight under the evaluated conditions, indicating an enhanced ability to localize low-abundance melanocyte signals within heterogeneous skin tissue. Following treatment group, the decline in melanocyte-associated signals demonstrated directional congruence with the outcomes of prior single-cell and ST characterization of the identical SKH-1 photoaging model [14]. The findings of this study collectively resulted in the extension of WDGTA from cell-type composition estimation to the spatial characterization of microenvironmental changes associated with UVR and treatment.

Nevertheless, the present study had several limitations. Firstly, WDGTA is contingent upon the quality and annotations of the scRNA-seq reference. Secondly, the graph is principally constructed from expression similarity, without full incorporation of spatial coordinates, or histological information. Thirdly, although feature-order perturbation demonstrated only a modest effect, the influence of gene-feature ordering and other DWT settings requires further investigation.

In summary, WDGTA provides an integrated spatial deconvolution framework that demonstrated robust aggregate performance and recovered anatomically concordant cell-type distributions across 10× Genomics Visium and Slide-seq V2 data. Its application to UVR-exposed skin further highlighted its potential to resolve low-abundance cell populations and characterize spatial microenvironmental responses associated with tissue injury and drug intervention.

**Key Points**

- WDGTA integrates wavelet-based multiscale denoising and reconstruction module, random projection forest graph construction, and graph attention for spatial transcriptomic deconvolution.
- WDGTA achieved the optimal performance across five benchmark datasets compared with seven established methods.
- Ablation analyses demonstrated the complementary contributions of wavelet denoising, graph construction, and semi-supervised learning.
- WDGTA preserved biologically relevant cortical and skin organization and improved the localization of low-abundance melanocyte signals in ultraviolet radiation-exposed skin.

## Supplementary Material

Supplementary_materials_bbag584

## Data Availability

Datasets used in this study can be downloaded from the Cell BLAST (https://cblast.gao-lab.org/), the 10× Genomics (https://www.10xgenomics.com/cn/resources/datasets). The mouse UVR-exposed skin dataset was provided by the Children’s Hospital of Zhejiang University School of Medicine and available upon reasonable request.
