# Supplementary material for: WDGTA: a wavelet-denoised graph attention framework for spatial transcriptomics deconvolution
Source: Brief Bioinform. 2026 Oct 19;27(5):bbag584. doi: 10.1093/bib/bbag584 (PMC13679754; doi:10.1093/bib/bbag584)
Supplement: Supplementary file 1 [file supplementary_materials_bbag584.docx]

**Supplementary Information**

*WDGTA: a Wavelet-Denoised Graph Attention Framework for Spatial Transcriptomics Deconvolution*

**Supplementary Note S1. Leakage-control audits for WDGTA**

**Rationale and evaluation setting**

WDGTA uses a transductive semi-supervised graph-learning setting in which the expression features of held-out nodes may participate in graph-based message passing while their cell-type proportion labels remain masked. Two complementary audits were performed to examine distinct potential routes of information leakage: reuse of the same annotated source cells across development and held-out pools, and unintended dependence of model optimisation or prediction on held-out labels.

**Source-cell identity audit**

Source-cell assignments were audited using the split file associated with the formal Private_skin_pseudo benchmark. The available artifact contained 5,874 training source cells and 1,468 test source cells. Source-cell identifiers were converted to sets and compared directly across partitions. No source-cell identity was shared between the training and test pools, and no duplicated cell assignment was detected. The available source-cell audit artifact contained training and test assignments; therefore, this result specifically supports separation of those two pools.

**Held-out-label permutation audit**

A separate execution audit tested whether held-out labels were accessed by the WDGTA training and prediction code path. The existing serialised UVR6 model input contained 3,175 held-out prediction nodes and six cell-type classes. The rows of the held-out label matrix were randomly permuted, while node features, graph structure, training labels, hyperparameters, model seed (123), and all other model inputs were kept unchanged. Baseline and permuted-label runs were each executed for two epochs. Data-label alignment checks passed before execution, and the original and permuted input files had different SHA-256 hashes, confirming that the held-out label artifact had been altered.

Training-metric logs, model checkpoints, and final prediction matrices were compared between the two runs. The training metrics were exactly equal, all 97 checkpoint tensors had identical values, and the final predictions were exactly equal. The maximum absolute numerical difference was 0 for each comparison. The baseline and permuted runs also produced identical SHA-256 hashes for the result and metrics files. These findings show that, under the audited execution setting, changing the held-out labels did not alter optimisation, learned parameters, or predictions.

**Scope of inference**

The two audits address complementary leakage routes. The source-cell audit supports disjoint training and test source-cell pools in the formal Private_skin_pseudo preparation artifact, whereas the held-out-label permutation audit supports the absence of held-out-label dependence in the audited WDGTA execution path. Because the two checks used complementary artifacts, they should not be interpreted as a single end-to-end rerun of the formal Private_skin_pseudo benchmark. In addition, the presence of held-out node features in a transductive graph is part of the stated evaluation design and is distinct from access to held-out labels.

**Supplementary Table S1. Results of the source-cell and held-out-label leakage-control audits.**

| **Audit** | **Artifact or comparison** | **Observed result** | **Interpretation** |
| --- | --- | --- | --- |
| Source-cell partition | Private_skin_pseudo source-cell split | Training: 5,874 cells; test: 1,468 cells; train-test overlap: 0; duplicate assignments: 0 | No source-cell reuse was detected between the audited training and test pools. |
| Input alignment | UVR6 serialised execution input | Training, validation, test, and prediction data-label alignment checks all passed | The label perturbation was applied to an aligned input artifact. |
| Held-out label perturbation | 3,175 prediction nodes; 6 classes | Held-out label rows were permuted; 0.8504% of rows remained unchanged by chance; input SHA-256 hashes differed | The held-out label artifact was materially altered while other model inputs were fixed. |
| Training metrics | Baseline versus permuted-label run | Exactly equal; maximum absolute numerical difference = 0 | Training trajectories did not depend on the held-out labels in the audited code path. |
| Model checkpoints | 97 checkpoint tensors | Variable manifests and tensor values were exactly equal; maximum absolute tensor difference = 0 | The learned parameters were unaffected by held-out-label permutation. |
| Final predictions | Baseline versus permuted-label result matrices | Exactly equal; maximum absolute numerical difference = 0 | Predictions were unaffected by held-out-label permutation. |
| Output-file verification | Result and metrics files | Result SHA-256: 4d8533c4935f4e8df5106087bc5a08ebf7eaa5332ffe682db57b0ab88cd5820f; metrics SHA-256: 809c8388733e45b24a1eca8982eb24aa7d38e16fb54acdc99bf76ecc37f0429b for both runs | Independent file-level verification confirmed identical outputs. |

*Note: The label-dependency experiment was limited to two epochs because its purpose was to test computational dependence on held-out labels rather than to estimate predictive performance. A passed audit indicates that no evidence of the specified leakage route was detected under the audited setting.*
